# 783 Understanding Barriers to the Implementation of Negative Pressure Wound Therapy in Acute Pediatric Burn Care

**DOI:** 10.1093/jbcr/irae036.324

**Published:** 2024-04-17

**Authors:** Maleea Holbert, Bronwyn R Griffin, Fiona M Wood, Roy M Kimble, Andrew J A Holland, Warwick Teague, Cody Frear, Dianne Crellin, Natalie Phillips, Kristen Storey, Yvonne M Singer, Leila Cuttle, Pauline Calleja, Jed Duff

**Affiliations:** Queensland Children's Hospital, South Brisbane, Queensland; Griffith University, Brisbane, Queensland; Fiona Wood Foundation, Perth, Western Australia; Queensland Children's Hospital, Brisbane, Queensland; The University of Sydney, Westmead, New South Wales; The Royal Children’s Hospital, Melbourne, Melbourne, Victoria; The University of Queensland, Brisbane, Queensland; Queensland Children's Hospital, Brisbane, Queensland; Griffith University, Melbourne, Victoria; Queensland University of Technology, South Brisbane, Queensland; Central Queensland University, Cairns, Queensland; Queensland University of Technology, Brisbane, Queensland; Queensland Children's Hospital, South Brisbane, Queensland; Griffith University, Brisbane, Queensland; Fiona Wood Foundation, Perth, Western Australia; Queensland Children's Hospital, Brisbane, Queensland; The University of Sydney, Westmead, New South Wales; The Royal Children’s Hospital, Melbourne, Melbourne, Victoria; The University of Queensland, Brisbane, Queensland; Queensland Children's Hospital, Brisbane, Queensland; Griffith University, Melbourne, Victoria; Queensland University of Technology, South Brisbane, Queensland; Central Queensland University, Cairns, Queensland; Queensland University of Technology, Brisbane, Queensland; Queensland Children's Hospital, South Brisbane, Queensland; Griffith University, Brisbane, Queensland; Fiona Wood Foundation, Perth, Western Australia; Queensland Children's Hospital, Brisbane, Queensland; The University of Sydney, Westmead, New South Wales; The Royal Children’s Hospital, Melbourne, Melbourne, Victoria; The University of Queensland, Brisbane, Queensland; Queensland Children's Hospital, Brisbane, Queensland; Griffith University, Melbourne, Victoria; Queensland University of Technology, South Brisbane, Queensland; Central Queensland University, Cairns, Queensland; Queensland University of Technology, Brisbane, Queensland; Queensland Children's Hospital, South Brisbane, Queensland; Griffith University, Brisbane, Queensland; Fiona Wood Foundation, Perth, Western Australia; Queensland Children's Hospital, Brisbane, Queensland; The University of Sydney, Westmead, New South Wales; The Royal Children’s Hospital, Melbourne, Melbourne, Victoria; The University of Queensland, Brisbane, Queensland; Queensland Children's Hospital, Brisbane, Queensland; Griffith University, Melbourne, Victoria; Queensland University of Technology, South Brisbane, Queensland; Central Queensland University, Cairns, Queensland; Queensland University of Technology, Brisbane, Queensland; Queensland Children's Hospital, South Brisbane, Queensland; Griffith University, Brisbane, Queensland; Fiona Wood Foundation, Perth, Western Australia; Queensland Children's Hospital, Brisbane, Queensland; The University of Sydney, Westmead, New South Wales; The Royal Children’s Hospital, Melbourne, Melbourne, Victoria; The University of Queensland, Brisbane, Queensland; Queensland Children's Hospital, Brisbane, Queensland; Griffith University, Melbourne, Victoria; Queensland University of Technology, South Brisbane, Queensland; Central Queensland University, Cairns, Queensland; Queensland University of Technology, Brisbane, Queensland; Queensland Children's Hospital, South Brisbane, Queensland; Griffith University, Brisbane, Queensland; Fiona Wood Foundation, Perth, Western Australia; Queensland Children's Hospital, Brisbane, Queensland; The University of Sydney, Westmead, New South Wales; The Royal Children’s Hospital, Melbourne, Melbourne, Victoria; The University of Queensland, Brisbane, Queensland; Queensland Children's Hospital, Brisbane, Queensland; Griffith University, Melbourne, Victoria; Queensland University of Technology, South Brisbane, Queensland; Central Queensland University, Cairns, Queensland; Queensland University of Technology, Brisbane, Queensland; Queensland Children's Hospital, South Brisbane, Queensland; Griffith University, Brisbane, Queensland; Fiona Wood Foundation, Perth, Western Australia; Queensland Children's Hospital, Brisbane, Queensland; The University of Sydney, Westmead, New South Wales; The Royal Children’s Hospital, Melbourne, Melbourne, Victoria; The University of Queensland, Brisbane, Queensland; Queensland Children's Hospital, Brisbane, Queensland; Griffith University, Melbourne, Victoria; Queensland University of Technology, South Brisbane, Queensland; Central Queensland University, Cairns, Queensland; Queensland University of Technology, Brisbane, Queensland; Queensland Children's Hospital, South Brisbane, Queensland; Griffith University, Brisbane, Queensland; Fiona Wood Foundation, Perth, Western Australia; Queensland Children's Hospital, Brisbane, Queensland; The University of Sydney, Westmead, New South Wales; The Royal Children’s Hospital, Melbourne, Melbourne, Victoria; The University of Queensland, Brisbane, Queensland; Queensland Children's Hospital, Brisbane, Queensland; Griffith University, Melbourne, Victoria; Queensland University of Technology, South Brisbane, Queensland; Central Queensland University, Cairns, Queensland; Queensland University of Technology, Brisbane, Queensland; Queensland Children's Hospital, South Brisbane, Queensland; Griffith University, Brisbane, Queensland; Fiona Wood Foundation, Perth, Western Australia; Queensland Children's Hospital, Brisbane, Queensland; The University of Sydney, Westmead, New South Wales; The Royal Children’s Hospital, Melbourne, Melbourne, Victoria; The University of Queensland, Brisbane, Queensland; Queensland Children's Hospital, Brisbane, Queensland; Griffith University, Melbourne, Victoria; Queensland University of Technology, South Brisbane, Queensland; Central Queensland University, Cairns, Queensland; Queensland University of Technology, Brisbane, Queensland; Queensland Children's Hospital, South Brisbane, Queensland; Griffith University, Brisbane, Queensland; Fiona Wood Foundation, Perth, Western Australia; Queensland Children's Hospital, Brisbane, Queensland; The University of Sydney, Westmead, New South Wales; The Royal Children’s Hospital, Melbourne, Melbourne, Victoria; The University of Queensland, Brisbane, Queensland; Queensland Children's Hospital, Brisbane, Queensland; Griffith University, Melbourne, Victoria; Queensland University of Technology, South Brisbane, Queensland; Central Queensland University, Cairns, Queensland; Queensland University of Technology, Brisbane, Queensland; Queensland Children's Hospital, South Brisbane, Queensland; Griffith University, Brisbane, Queensland; Fiona Wood Foundation, Perth, Western Australia; Queensland Children's Hospital, Brisbane, Queensland; The University of Sydney, Westmead, New South Wales; The Royal Children’s Hospital, Melbourne, Melbourne, Victoria; The University of Queensland, Brisbane, Queensland; Queensland Children's Hospital, Brisbane, Queensland; Griffith University, Melbourne, Victoria; Queensland University of Technology, South Brisbane, Queensland; Central Queensland University, Cairns, Queensland; Queensland University of Technology, Brisbane, Queensland; Queensland Children's Hospital, South Brisbane, Queensland; Griffith University, Brisbane, Queensland; Fiona Wood Foundation, Perth, Western Australia; Queensland Children's Hospital, Brisbane, Queensland; The University of Sydney, Westmead, New South Wales; The Royal Children’s Hospital, Melbourne, Melbourne, Victoria; The University of Queensland, Brisbane, Queensland; Queensland Children's Hospital, Brisbane, Queensland; Griffith University, Melbourne, Victoria; Queensland University of Technology, South Brisbane, Queensland; Central Queensland University, Cairns, Queensland; Queensland University of Technology, Brisbane, Queensland; Queensland Children's Hospital, South Brisbane, Queensland; Griffith University, Brisbane, Queensland; Fiona Wood Foundation, Perth, Western Australia; Queensland Children's Hospital, Brisbane, Queensland; The University of Sydney, Westmead, New South Wales; The Royal Children’s Hospital, Melbourne, Melbourne, Victoria; The University of Queensland, Brisbane, Queensland; Queensland Children's Hospital, Brisbane, Queensland; Griffith University, Melbourne, Victoria; Queensland University of Technology, South Brisbane, Queensland; Central Queensland University, Cairns, Queensland; Queensland University of Technology, Brisbane, Queensland; Queensland Children's Hospital, South Brisbane, Queensland; Griffith University, Brisbane, Queensland; Fiona Wood Foundation, Perth, Western Australia; Queensland Children's Hospital, Brisbane, Queensland; The University of Sydney, Westmead, New South Wales; The Royal Children’s Hospital, Melbourne, Melbourne, Victoria; The University of Queensland, Brisbane, Queensland; Queensland Children's Hospital, Brisbane, Queensland; Griffith University, Melbourne, Victoria; Queensland University of Technology, South Brisbane, Queensland; Central Queensland University, Cairns, Queensland; Queensland University of Technology, Brisbane, Queensland

## Abstract

**Introduction:**

Pediatric burn injuries pose a major clinical problem worldwide and result in significant morbidity. Early application of adjunctive negative pressure wound therapy (NPWT) has been shown to significantly improve time to re-epithelialization in pediatric burn patients, however this treatment has not yet been reliably or consistently adopted.

**Methods:**

This investigation used a sequential mixed methods design to identify and explore barriers to the implementation of adjunctive NPWT in acute pediatric burn care. An online questionnaire was developed and disseminated to healthcare professionals within four major pediatric hospitals, each with a dedicated burns service. Specific barrier data were coded according to the Consolidated Framework for Implementation Research (CFIR). Semi-structured interviews were then conducted with senior clinicians across the four participating hospitals to adapt and tailor implementation strategies to local contexts. A stakeholder consensus meeting was then conducted to consolidate implementation strategies and local processes.

**Results:**

A total of 63 healthcare professionals participated in the online questionnaire, and semi-structured interviews were conducted with nine senior burn clinicians. Two interviews were also conducted with parents and caregivers of pediatric burn patients who had received NPWT as part of their acute burn treatment within the last 12-months. This investigation identified eight implementation barriers across all five CFIR domains then co-designed targeted strategies to address these identified barriers. Barriers included lack of available resources, limited access to knowledge and information, individual stage of change, patient needs and resources, limited knowledge and beliefs about the intervention, lack of external policies and incentives, intervention complexity, and planning.

**Conclusions:**

There are multiple and inter-related contextual characteristics that influence the uptake of adjunctive NPWT into acute pediatric burn settings. In order to implement NPWT into clinical practice for the acute treatment of pediatric burn injuries, additional resources, education, training, and updates to policies and guidelines are required. It is anticipated that NPWT, in conjunction with tailored implementation strategies, will enhance adoption and sustainability.

**Applicability of Research to Practice:**

The time lag in evidence-to-practice implementation is a well-recognized issue in clinical and healthcare research. This investigation is one of the first to define barriers and enablers of implementation in acute pediatric burns. Findings from this research can help inform and guide other acute burn related implementation studies in the future, the implementation of other technologies, devices, or treatment pathways for pediatric patients in a healthcare setting.

